# GC-FID quantification of methanol, ethanol, 2-propanol, acetone, ethylene glycol, diethylene glycol, 1,2-propylene glycol and 1,3-propylene glycol in plasma

**DOI:** 10.1093/jat/bkag013

**Published:** 2026-02-15

**Authors:** Mohsin El Amrani, Arjan Zandbergen, Mike Groot, Tim Bognar, Erin H Smeijsters, Kim C M van der Elst

**Affiliations:** Department of Clinical Pharmacy, Division Laboratories, Pharmacy and Biomedical Genetics, University Medical Center Utrecht, Utrecht University, Utrecht, 3508 GA, The Netherlands; Department of Clinical Pharmacy, Division Laboratories, Pharmacy and Biomedical Genetics, University Medical Center Utrecht, Utrecht University, Utrecht, 3508 GA, The Netherlands; Department of Clinical Pharmacy, Division Laboratories, Pharmacy and Biomedical Genetics, University Medical Center Utrecht, Utrecht University, Utrecht, 3508 GA, The Netherlands; Department of Clinical Pharmacy, Division Laboratories, Pharmacy and Biomedical Genetics, University Medical Center Utrecht, Utrecht University, Utrecht, 3508 GA, The Netherlands; Department of Clinical Pharmacy, Division Laboratories, Pharmacy and Biomedical Genetics, University Medical Center Utrecht, Utrecht University, Utrecht, 3508 GA, The Netherlands; Department of Clinical Pharmacy, Division Laboratories, Pharmacy and Biomedical Genetics, University Medical Center Utrecht, Utrecht University, Utrecht, 3508 GA, The Netherlands

## Abstract

Alcohol and glycol ingestion, including substances such as methanol, ethylene glycol, and isopropanol, constitute a serious medical emergency that requires prompt diagnosis and treatment of patients. Rapid and accurate quantification of these compounds in plasma is essential to guide clinical decision-making and prevent delays in treatment that could result in irreversible organ damage or death. Many healthcare facilities lack analytical methods capable of simultaneously quantifying both alcohols and glycols in a single run. This study describes the development and validation of a rapid gas chromatography–flame ionization detection (GC-FID) method for the simultaneous screening and quantification of methanol, ethanol, 2-propanol, acetone, ethylene glycol, diethylene glycol, 1,2-propylene glycol, and 1,3-propylene glycol in human plasma. Plasma samples were prepared using a protein precipitation technique with acetonitrile containing two internal standards: 2-butanol and 1,4-butanediol. Acetonitrile effectively precipitated plasma proteins. The supernatant was then subjected to GC-FID analysis for quantification of the target al.ohols and glycols. The total analytical run time was 5 minutes, enabling the quantification of eight analytes in a single injection. The method demonstrated excellent linearity, with correlation coefficients (*R*^2^) exceeding 0.9995 for all compounds. The linear dynamic range was 40–1280 mg/L for methanol, 2-propanol, acetone, ethylene glycol, diethylene glycol, 1,2-propylene glycol, and 1,3-propylene glycol, and 80–2560 mg/L for ethanol. Within-run and between-run precision and accuracy (CV and bias) for all analytes were within the predefined acceptance criteria of ±15%. No significant interference, carry-over, or matrix effects were observed, confirming the method’s selectivity and robustness. The developed GC-FID method enables rapid, accurate, and simultaneous quantification of toxic alcohols and glycols in plasma within a 5-minute run time. The excellent linearity, precision, and selectivity of the method met al. analytical performance criteria, making it well-suited for routine clinical use. This method provides a valuable tool for timely diagnosis and management of suspected toxic alcohol and glycol ingestions in patients in emergency settings.

## Introduction

Ingestion of toxic alcohols and glycols, including methanol, ethylene glycol, isopropanol, and diethylene glycol, is a medical emergency associated with significant morbidity and mortality in patients [1, 2]. These compounds are found in common consumer products, including as additives in cleaning solutions (methanol), rubbing alcohol (2-propanol), nail polish remover (acetone), and antifreeze (glycols), and are metabolized into toxic intermediates, such as formic acid and oxalic acid, which can lead to severe metabolic acidosis, central nervous system depression, and multi-organ damage [3–5]. Without timely diagnosis and treatment, outcomes can include irreversible blindness, acute kidney injury, or death [6].

Clinical symptoms are often nonspecific, and while surrogate markers such as anion gap and osmol gap may support a ­diagnosis, they lack sensitivity and specificity [3]. Therefore, direct measurement of the parent compounds in plasma remains the gold standard for confirmation of toxic alcohol or glycol exposure and for determining the need for additional treatment, such as antidotes and hemodialysis.

Access to a comprehensive and rapid analytical method is often limited in clinical settings, as logistical and analytical challenges may delay the timely availability of results. Many laboratories are not equipped with techniques that can simultaneously identify and quantify a wide range of alcohols and glycols in a timely manner. This can delay administration of antidotes (e.g. ethanol and fomepizole) or initiation of hemodialysis, increasing the risk of complications [7].

Gas chromatography with flame ionization detection (GC-FID) is a robust, cost-effective, and widely accessible method for the analysis of volatile and semi-volatile compounds. It is well-suited for clinical toxicology applications due to its high sensitivity, reproducibility, and ability to quantify multiple analytes with minimal sample preparation [8]. Nevertheless, current gas chromatographic methods typically suffer from extended analysis times, labor-intensive derivatization steps, restricted applicability to only a subset of relevant compounds, or require the use of two different analytical columns [9–15].

In this study, we describe the development and validation of a rapid and robust 5-minute GC-FID method for the simultaneous quantification of methanol, ethanol, 2-propanol, acetone, ethylene glycol, diethylene glycol, 1,2-propylene glycol, and 1,3-propylene glycol in human plasma. Using a simple protein precipitation step with internal standards, injected on a single column, without prior derivatization. This method offers an efficient and reliable tool for use in emergency toxicology, supporting a fast and accurate diagnosis of suspected poisonings.

## Materials and methods

### Chemicals and reagents

Methanol was obtained from Biosolve (Valkenswaard, The Netherlands), ethanol from Merck (Darmstadt, Germany), and acetone, 2-propanol, ethylene glycol, diethylene glycol, and 1,2-propylene glycol were purchased from Sigma-Aldrich (St. Louis, MO, USA). 1,3-Propylene glycol was obtained from Merck. Acetonitrile (HPLC grade) was acquired from Biosolve. The internal standards 2-butanol and 1,4-butanediol were both purchased from Sigma-Aldrich. All reagents were of analytical or HPLC grade.

### Preparation of standards, internal standards, and quality controls

A working calibration standard (Std 6) was prepared in water with the following final concentrations: ethanol at 2560 mg/L and methanol, acetone, 2-propanol, ethylene glycol, diethylene glycol, 1,2-propylene glycol, and 1,3-propylene glycol at 1280 mg/L. To prepare Std 6, the following volumes of 80 g/L stock solutions were added to a 50 mL volumetric flask containing approximately 20 mL of water: 1.60 mL ethanol and 0.80 mL each of methanol, acetone, 2-propanol, ethylene glycol, diethylene glycol, 1,2-propylene glycol, and 1,3-propylene glycol. During pipetting, approximately 1 mL of water was intermittently added using a Pasteur pipette to rinse any residual solution into the flask. The flask was kept covered with a glass stopper as much as possible to minimize evaporation. After all components were added, the volume was adjusted to the 50 mL mark with water and mixed thoroughly. The lower calibration standards (Std 1–Std 5) were prepared by serial 1:2 dilutions of Std 6. For each dilution, 25 mL of the preceding standard was mixed with 25 mL of water in a 50 mL volumetric flask and diluted to volume. The final ethanol standard concentrations were 80–2560 mg/L (80, 160, 320, 640, 1280, 2560), and for methanol, acetone, 2-propanol, ethylene glycol, diethylene glycol, 1,2-propylene glycol, and 1,3-propylene glycol, they were 40–1280 mg/L (40, 80, 160, 320, 640, 1280). The internal standard (IS) solution was prepared by pipetting 50 µL of 2-butanol and 50 µL of 1,4-butanediol into a 250 mL volumetric flask containing approximately 100 mL of acetonitrile (Biosolve), to achieve a concentration of approximately 200 mg/L. The flask was then filled to the mark with additional acetonitrile and mixed thoroughly to ensure complete homogenization. Quality control (QC) samples at the lower limit of quantification (LLOQ) and low, medium, and high concentrations were prepared independently from the calibration standards using separate 80 g/L stock solutions for each analyte. The target concentrations for the QC samples were as follows: 80, 240, 800, and 2000 mg/L for ethanol and 40, 120, 400, and 1000 mg/L for methanol, acetone, 2-propanol, ethylene glycol, diethylene glycol, 1,2-propylene glycol, and 1,3-propylene glycol, respectively.

### Instrumentation and chromatographic conditions

Sample mixing was performed using a multi-tube vortex mixer DVX-2500 from VWR (Pennsylvania, USA). Sample centrifugation was performed on a Mikro 220 from Hettich (Kirchlengern, Germany). Gas chromatography analysis was conducted on a TRACE 1600 GC-FID system from Interscience BV/Thermo Fisher Scientific (Breda, The Netherlands). Chromatographic separation was achieved using an RTx-502.2 capillary column (30 m length × 0.32 mm internal diameter × 1.8 µm film thickness) from Restek (Pennsylvania, USA). The injection volume was 0.2 µL, with the injector maintained at 300 °C and operated in split mode using a split ratio of 5:1. The carrier gas was helium, with a constant flow rate of 5 mL/min. The oven temperature program was as follows: Initial temperature: 40 °C, held for 1 minute, then ramp to 185 °C at 3.45 minutes, final ramp to 270 °C at 4.3 minutes, and hold till 5 minutes. The flame ionization detector (FID) was maintained at 300 °C with the following gas flows: Air: 350 mL/min, hydrogen: 35 mL/min, and makeup gas (helium): 40 mL/min.

### Sample preparation for GC-FID analysis

In a glass vial, 250 µL of either standard, patient plasma sample, or QC material was pipetted, followed by the addition of 250 µL of IS solution containing 2-butanol and 1,4-butanediol (each at 200 mg/L in acetonitrile). Vials were sealed tightly and mixed thoroughly using a vortex mixer at 2500 RPM for 1 minute to ensure complete protein precipitation. After mixing, the samples were centrifuged at 11290 G for 5 minutes. A 0.2 µL aliquot of the clear supernatant was injected into the GC-FID system for analysis.

### Evaluation of internal standard performance

The effect of the IS selection on the quantification of ethylene glycol and methanol was evaluated by comparing 2-butanol and 1,4-butanediol as IS. A total of 50 consecutive injections of the QC medium sample, prepared according to the sample preparation protocol, were analyzed using GC-FID. For each injection, the peak area ratio of ethylene glycol and methanol to IS was calculated. The variability in response ratio was assessed by comparing the relative standard deviations of the ethylene glycol and methanol/IS when using 2-butanol versus 1,4-butanediol. The IS yielding the lowest variability was considered more suitable for quantification.

### Evaluation of injection volume on signal intensity and resolution

The effect of varying injection volumes on signal intensity and chromatographic resolution was assessed using the QC Low sample, prepared as described in the sample preparation section. Injections of 0.1, 0.2, and 0.3 µL were performed under identical chromatographic conditions, with the injector operated in split mode at a split ratio of 5:1. Each injection volume was evaluated for its impact on peak height, peak area, and resolution between neighboring analytes. The goal was to determine the optimal injection volume that provided sufficient signal intensity without compromising peak shape or chromatographic separation.

### Evaluation of liner contamination and carry-over

To evaluate the effect of liner contamination on carry-over, particularly for glycol analytes, a freshly installed inlet liner was used to perform 30 consecutive injections of the plasma QC High sample, prepared as described in the sample preparation section. Following each plasma injection, a blank water: acetonitrile (50:50, v/v) sample was injected to assess potential carry-over. Carry-over was assessed by monitoring the presence and intensity of analyte peaks in the blank injections following each QC high sample. The threshold for unacceptable carry-over was defined as a peak area exceeding 20% of the signal observed at the lower limit of quantification (LLOQ). The goal of this experiment was to determine the maximum number of plasma sample injections that could be performed on a single liner before carryover exceeded this threshold.

### Method validation

The analytical method was validated in accordance with the *ICH guideline M10 on bioanalytical method validation and study sample analysis* [16]. Method performance was assessed in terms of linearity, accuracy, precision, selectivity, matrix effects, and autosampler stability. Linearity was evaluated across the validated calibration range for each analyte. Autosampler stability was assessed by reinjecting QC Low and QC High samples after 24 hours of storage in the autosampler at room temperature. Accuracy and precision were evaluated, both within-run and between-run, by analyzing QC samples (LLOQ, Low, Medium, and High) in five replicates over three separate days. Selectivity was assessed by analyzing 6 different blank human plasma samples to confirm the absence of interfering peaks at the retention times of the target analytes. Any peak detected at the analyte retention time was integrated, and its signal was compared to the corresponding LLOQ signal (expressed as a percentage). Specificity was tested by analyzing a gamma hydroxybutyric acid (GHB) solution of 500 mg/L dissolved in water. The matrix effect was evaluated by spiking 6 individual blank human plasma samples at QC low and QC high concentrations, then analyzing the variability. Stability was evaluated over a 3-day period at both room temperature and refrigerated conditions using QC low and high concentrations.

## Results and discussion

### Evaluation of internal standard performance

The boiling points of glycols are at least 100 °C higher than those of alcohols, resulting in markedly different behaviors within the GC inlet system. These differences influence vaporization efficiency, split ratio consistency, and susceptibility to carry-over, all of which can contribute to variability in the amount of analyte delivered to the capillary column. To address this, two IS, 2-butanol and 1,4-butanediol, were evaluated for their ability to compensate for such variability, particularly in the quantification of diols and alcohols. Ethylene glycol was selected to represent the -diol analytes and methanol the alcohol analytes, as these two compounds exhibit distinct behaviors during analysis, including tailing and carry-over. The performance of the two IS solutions was compared based on precision and consistency over 50 consecutive injections of a QC medium sample, with the goal of identifying the IS that provided the lowest analytical variability and best correction for inlet-related effects.

**Figure 1:**
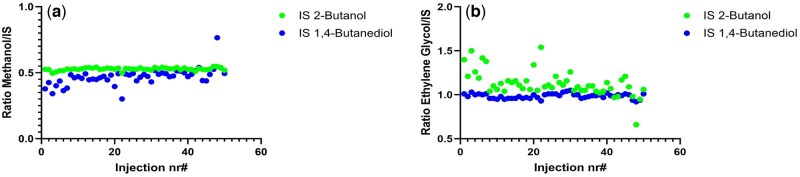
Comparison of internal the standard correction for methanol (a) and ethylene glycol (b) using either 2-butanol or 1,4-butanediol across 50 consecutive injections of a QC medium sample.

1,4-Butanediol provided the lowest variability in results for ethylene glycol, whereas 2-butanol showed the lowest variability for methanol, as demonstrated in Fig. 1a and b. This outcome is likely attributable to structural similarities and physicochemical properties, particularly the presence of hydroxyl groups and comparable boiling points. Such similarities may contribute to more consistent behavior during vaporization and injection, enhancing the suitability of 1,4-butanediol as an IS for glycol analytes. For the more volatile analytes, 2-butanol provided the lowest variability between consecutive injections (data not shown).

### Evaluation of injection volume on signal intensity and resolution

**Figure 2:**
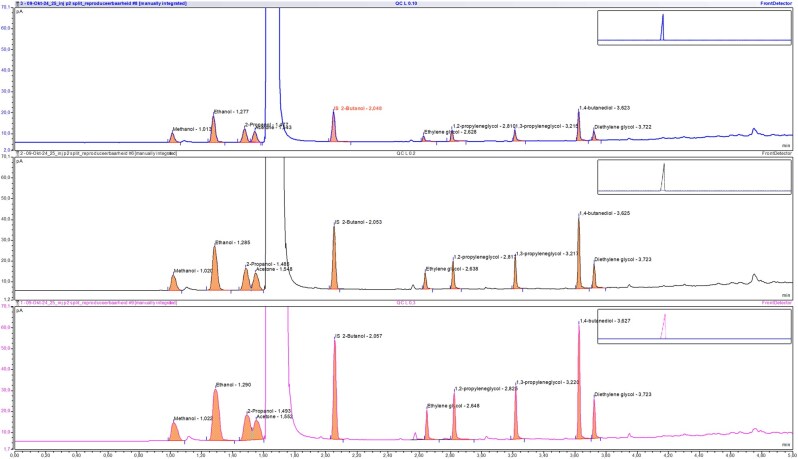
GC-FID chromatograms obtained at different injection volumes showing the separation of methanol, ethanol, 2-propanol, acetone, ethylene glycol, 1,2-propylene glycol, 1,3-propylene glycol, diethylene glycol, and the internal standards (2-butanol and 1,4-butanediol). An injection volume of 0.2 µL (middle chromatogram) provided a good peak shape, optimal separation between 2-propanol and acetone, and an acceptable signal to noise ratio for ethylene glycol compared to higher and lower injection volumes (bottom and top chromatograms, respectively).

Injection volume and split ratio are interdependent parameters that influence chromatographic performance. To evaluate their impact, three different injection volumes of 0.1, 0.2, and 0.3 µL were tested at a fixed split ratio of 5:1. The goal was to identify the optimal injection volume that provides sufficient signal intensity while maintaining peak resolution and symmetry. An injection volume of 0.2 µL provided an optimal balance between chromatographic resolution, particularly for 2-propanol and acetone, peak shape, and an acceptable signal-to-noise ratio for ethylene glycol (Fig. 2).

**Figure 3:**
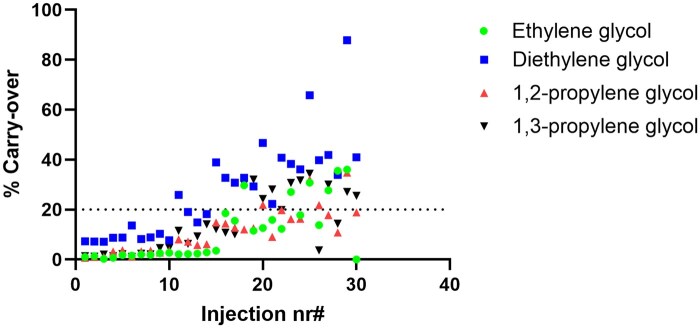
Carry-over percentage for ethylene glycol, diethylene glycol, 1,2-propylene glycol, and 1,3-propylene glycol in relation to the number of injections performed. At 10 injections, carry-over for all components was within the acceptation criteria of 20%.

### Evaluation of liner contamination and carry-over

Remnant plasma proteins and lipids dissolved in the supernatant after sample preparation can contaminate the liner and act as a secondary stationary phase for analytes, particularly glycols since they have a much higher boiling point. This can lead to peak tailing and carry-over. To assess system robustness, a test was conducted to determine the maximum number of plasma sample injections that could be performed without significantly affecting peak shape while maintaining carry-over below the acceptable threshold of 20%. No carry-over was observed for methanol, ethanol, acetone, and 2-propanol over the 30 consecutive plasma and blank injections (data not shown). For ethylene glycol, 1,2-propylene glycol, and 1,3-propyleneglycol, acceptable carry-over values were maintained for up to approximately 15 plasma injections (Fig. 3). In contrast, for diethylene glycol, the maximum allowable number of plasma injections was limited to 10 to remain below the acceptable carry-over threshold. Therefore, the liner needs to be replaced after a maximum of 10 plasma sample injections; this excludes standards since they are diluted in water.

### Method validation

**Figure 4:**
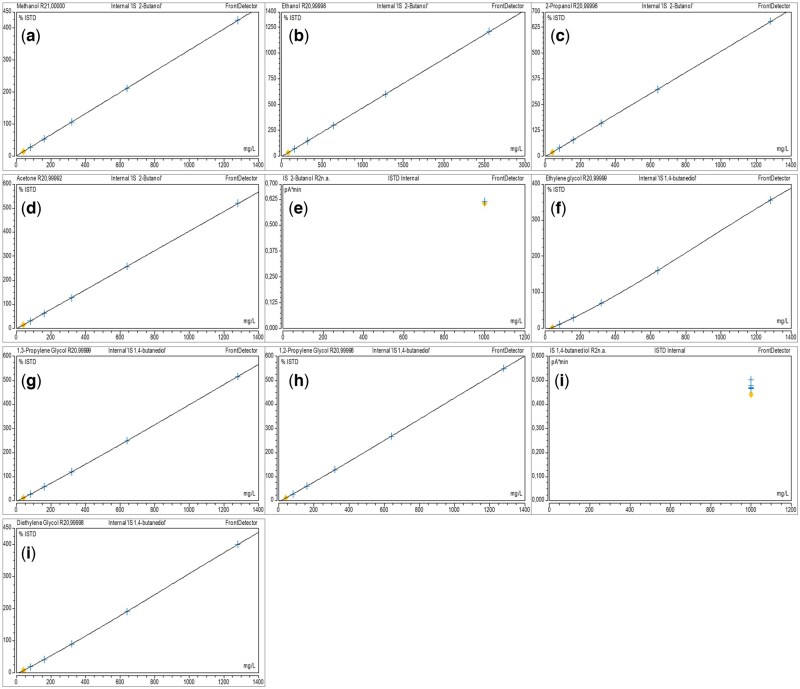
Calibration curves for a) methanol, b) ethanol, c) 2-propanol, d) acetone, f) ethylene glycol, g) 1,3-propylene glycol, h) 1,2-propylene glycol, and j) diethylene glycol and their internal standards, e) 2-butanol and i) 1,4-butanediol, *n* = 6 standard points.

**Figure 5:**
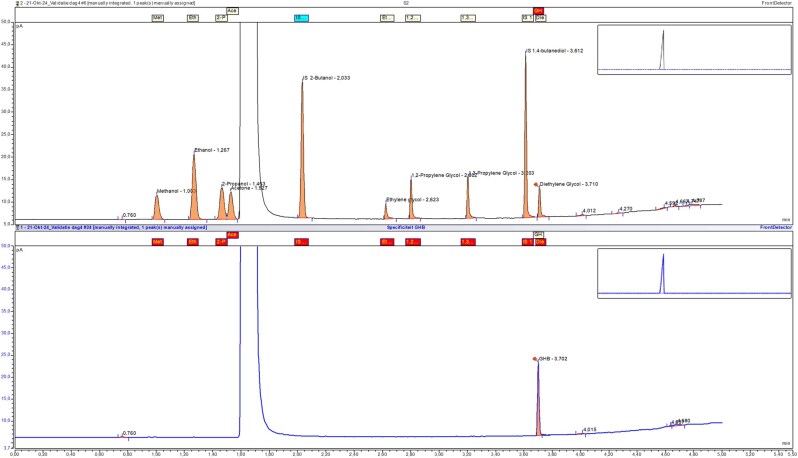
Specificity assessment of gamma-hydroxybutyric acid (GHB). The bottom chromatogram shows GHB with a retention time of 3.702 minutes, closely overlapping with diethylene glycol in the top chromatogram (retention time: 3.710 minutes; standard 2), indicating coelution under the current chromatographic conditions.

The GC-FID method demonstrated excellent performance across all validated analytes. A linear calibration model was applied for methanol, ethanol, 2-propanol, and acetone, while a second-order polynomial regression was used for ethylene glycol, diethylene glycol, 1,2-propylene glycol, and 1,3-propylene glycol to account for minor non-linearity at higher concentrations. All analytes showed excellent correlation within their respective models, with coefficients of determination (R^2^) exceeding 0.9999 (Fig. 4). Accuracy and precision were assessed at four QC levels: LLOQ, Low, Medium, and High. Intra- and inter-day precision (%CV) and accuracy (%Bias) were all within the ±15% acceptance criteria, consistent with ICH M10 guidelines. Full accuracy and precision results are presented in Table 1. Selectivity testing showed no interfering peaks at the retention times of the analytes in six blank matrix samples. All background signals were below 20% of the LLOQ response, confirming acceptable selectivity. Specificity testing identified co-elution of gamma-hydroxybutyric acid (GHB) with diethylene glycol under the current chromatographic conditions (Fig. 5). As a result, when a diethylene glycol peak is observed, a separate confirmatory test for GHB is necessary to ensure correct identification. No significant degradation of any analyte was observed under either condition, confirming the short-term stability of the compounds in plasma.

**Table 1:** Precision and accuracy: validation performed during 3 days using QC, LLOQ, low, medium, and high (*n* = 5) samples.

	LLOQ			Low			Medium			High		
QC	WR	BR	B	WR	BR	B	WR	BR	B	WR	BR	B
Methanol	0.4	1.2	3.7	0.9	1.2	0.4	0.4	1.2	3.7	0.6	1.6	0.2
Ethanol	1.0	5.9	−3.6	0.5	0.7	3.1	0.4	0.9	4.0	0.4	1.7	0.7
2-Propanol	1.2	3.9	−2.6	0.4	0.9	2.6	0.4	0.7	3.4	0.3	1.3	0.2
Aceton	2.0	13.5	−0.7	0.5	3.1	8.2	0.5	3.1	8.7	0.7	6.4	1.2
Ethylene glycol	1.9	5.7	10.1	3.3	4.4	−0.3	1.4	2.8	6.5	1.1	3.2	1.5
Diethylene glycol	1.0	6.3	5.3	2.1	3.5	−0.3	1.9	2.4	3.2	1.0	1.8	0.1
1,2-Propylene glycol	1.8	8.1	12.5	2.0	3.4	3.8	1.6	2.1	5.0	0.9	3.2	−1.2
1,3-Propylene glycol	2.0	5.4	1.9	2.7	3.8	0.9	1.2	2.1	4.3	1.1	4.1	−1.0

B, bias [%]; BR, between run CV [%]; LLOQ, lower limit of quantification; QC, quality control; WR, within run CV [%].

## Discussion and conclusion

This study describes the development and validation of a rapid and reliable GC-FID method for the simultaneous quantification of eight toxic alcohols and glycols: methanol, ethanol, 2-propanol, acetone, ethylene glycol, diethylene glycol, 1,2-propylene glycol, and 1,3-propylene glycol, in human plasma. The method is based on a simple protein precipitation protocol, using dual IS, and provides a total run time of only 5 minutes per sample. The method demonstrated excellent analytical performance, including linearity with an *R*^2^ > 0.9999 for all components, accuracy, and precision across a wide calibration range.

In cases of intoxication, plasma concentrations of certain analytes may exceed the upper limit of the validated calibration range. In such situations, clinical decision-making is not compromised, as concentrations exceeding the calibration range already indicate ­severe intoxication and the need for urgent interventions, such as hemodialysis. For analytical purposes, samples with concentrations above the calibration range are reanalyzed following dilution with reverse osmosis water to bring them within the validated range.

Analyte stability was confirmed for at least 3 days under both room temperature and refrigerated storage. Selectivity and specificity met the predefined validation criteria, although GHB was found to coelute with diethylene glycol, necessitating confirmatory testing in such cases. However, this overlap may be advantageous when no diethylene glycol peak is present, as the absence of this peak would also indicate that the patient is negative for GHB.

To maintain method integrity and prevent analyte carry-over, particularly for high-boiling-point glycols, it is necessary to replace the GC inlet liner after a maximum of 10 plasma sample injections. This operational safeguard ensures consistent chromatographic performance and reduces the risk of carry-over artifacts in clinical diagnostics. Although this requirement increases consumable use, this method is intended for emergency settings involving a limited number of patients, where rapid analysis is critical for clinical outcomes. Under these conditions, the associated costs remain acceptable.

One important methodological limitation is that, although rare, 1,4-butanediol can be a source of intoxication, being used due to its metabolism to GHB, and could interfere with the internal standard signal. In such cases, 1,4-butanediol is metabolized to GHB, which may generate an additional peak, coeluting near diethylene glycol [17]. This would be accompanied by an increase in the peak area of the internal standard and should be assessed during data analysis.

A polyethylene glycol–based column could offer improved separation of glycols, but its strong retention of alcohols would require longer temperature programs or a separate analytical run on a different column. In this study, a medium-polar column provided a better balance, allowing simultaneous analysis of glycols and alcohols in a single injection on a single column, with a total run time of only 5 minutes. This approach minimizes total analysis time, which is essential in emergency settings where rapid clinical decision-making is critical.

In conclusion, this GC-FID method offers a fast, accurate, and cost-effective solution for the routine quantification of toxic alcohols and glycols in emergency clinical settings, supporting a fast and accurate diagnosis of suspected poisonings in patients.

## References

[1] Inman B , Maddry JK, Ng PC et al. High risk and low prevalence diseases: toxic alcohol ingestion. Am J Emerg Med 2023;67:29–36. 36796238 10.1016/j.ajem.2023.01.048

[2] Latus J , Kimmel M, Alscher MD et al. Ethylene glycol poisoning: a rare but life-threatening cause of metabolic acidosis—a single-centre experience. Clin Kidney J 2012;5:120–3. 25503773 10.1093/ckj/sfs009 PMC4235595

[3] Kraut JA , Mullins ME. Toxic alcohols. N Engl J Med 2018;378:270–80. 29342392 10.1056/NEJMra1615295

[4] Nelson LS , Hoffman RS, Howland MA et al. Goldfrank’s Toxicologic Emergencies , 11th edn. New York, NY: McGraw-Hill, 2019.

[5] Barceloux DG , Randall Bond G, Krenzelok EP et al.; American Academy of Clinical Toxicology Ad Hoc Committee on the Treatment Guidelines for Methanol Poisoning. American Academy of Clinical Toxicology practice guidelines on the treatment of methanol poisoning. J Toxicol: Clin Toxicol 2002;40:415–46. 12216995 10.1081/clt-120006745

[6] Jacobsen D , McMartin KE. Methanol and ethylene glycol poisonings: mechanism of toxicity, clinical course, diagnosis and treatment. Med Toxicol 1986;1:309–34. 3537623 10.1007/BF03259846

[7] Jammalamadaka D , Raissi S. Ethylene glycol, methanol and isopropyl alcohol intoxication. Am J Med Sci 2010;339:276–81. 20090509 10.1097/MAJ.0b013e3181c94601

[8] Poole CF. Ionization-based detectors for gas chromatography. J Chromatogr A 2015;1421:137–53. 25757823 10.1016/j.chroma.2015.02.061

[9] Orton DJ , Boyd JM, Affleck D et al. One-step extraction and quantitation of toxic alcohols and ethylene glycol in plasma by capillary gas chromatography (GC) with flame ionization detection (FID). Clin Biochem 2016;49:132–8. 26385496 10.1016/j.clinbiochem.2015.09.007

[10] Williams RH , Shah SM, Maggiore JA et al. Simultaneous detection and quantitation of diethylene glycol, ethylene glycol, and the toxic alcohols in serum using capillary column gas chromatography. J Anal Toxicol 2000;24:621–6. 11043669 10.1093/jat/24.7.621

[11] Cheung S , Lin W. Simultaneous determination of methanol, ethanol, acetone, isopropanol and ethylene glycol in plasma by gas chromatography. J Chromatogr 1987;414:248–50. 3571391 10.1016/0378-4347(87)80049-x

[12] Yao HH , Porter WH. Simultaneous determination of ethylene glycol and its major toxic metabolite, glycolic acid, in serum by gas chromatography. Clin Chem 1996;42:292–7. 8595726

[13] Livesey JF , Perkins SL, Tokessy NE et al. Simultaneous determination of alcohols and ethylene glycol in serum by packed-or capillary-column gas chromatography. Clin Chem 1995;41:300–5. 7874784

[14] Gembus V , Goulle J, Lacroix C. Determination of glycols in biological specimens by gas chromatography-mass spectrometry. J Anal Toxicol 2002;26:280–5. 12166815 10.1093/jat/26.5.280

[15] Edstam M , Sundqvist G, Kronstrand R. Quantitation of alcohols and acetone in postmortem blood and urine using headspace gas chromatography mass spectrometry. J Anal Toxicol 2025 10.1093/jat/bkaf076. PMC13079083 40838999

[16] European Medicines Agency. *ICH Guideline M10 on Bioanalytical Method Validation and Study Sample Analysis*. https://www.ema.europa.eu/en/documents/scientific-guideline/ich-guideline-m10-bioanalytical-method-validation-step-5_en.pdf (17 July 2025, date last accessed).

[17] Dufayet L , Bargel S, Bonnet A et al. Gamma-hydroxybutyrate (GHB), 1, 4-butanediol (1, 4BD), and gamma-butyrolactone (GBL) intoxication: a state-of-the-art review. Regul Toxicol Pharmacol 2023;142:105435. 37343712 10.1016/j.yrtph.2023.105435

